# Cardiomiopatia Atrial e Hipertensão: Conexões entre Rigidez Arterial e Arritmias Atriais Subclínicas

**DOI:** 10.36660/abc.20240598

**Published:** 2024-10-22

**Authors:** Ronaldo Altenburg Gismondi, Mario Fritsch Neves

**Affiliations:** 1 Universidade Federal Fluminense Departamento de Medicina Clínica Niterói RJ Brasil Universidade Federal Fluminense - Departamento de Medicina Clínica, Niterói, RJ – Brasil; 2 Universidade do Estado do Rio de Janeiro Clinica Medica Rio de Janeiro RJ Brasil Universidade do Estado do Rio de Janeiro - Clinica Medica, Rio de Janeiro, RJ – Brasil

**Keywords:** Hipertensão, Rigidez Vascular, Arritmias Cardíacas

A hipertensão arterial sistêmica (HAS) é o principal fator de risco para doença cardiovascular, promovendo ativação do sistema renina-angiotensina-aldosterona (SRAA), disfunção endotelial, rigidez arterial, fibrose e hipertrofia miocárdica. Estudos prospectivos mostram que as consequências incluem hipertrofia ventricular esquerda (HVE), sobrecarga atrial esquerda (AE) e maior risco de fibrilação atrial (FA).^1^ No estudo clássico de Framingham, a hipertensão foi um preditor independente de FA e, por si só, foi responsável por mais casos dessa arritmia do que qualquer outro fator de risco.^1^

O reconhecimento de uma fase "pré-FA", onde as alterações funcionais atriais ainda não se traduziram em dilatação ou FA clinicamente manifesta, é de suma importância na cardiologia moderna, pois o reconhecimento precoce permite intervenções terapêuticas antes que a condição evolua para uma doença estabelecida. Alterações funcionais e estruturais no AE são preditores de arritmias atriais durante o monitoramento do ritmo, e estas, por sua vez, são preditores de um risco maior de FA.^2,3^

A cardiomiopatia atrial é uma condição cada vez mais reconhecida, identificada por meio de vários testes funcionais, e o método mais utilizado é a ecocardiografia. No passado, a medição do volume do AE era a principal medida de disfunção atrial e, portanto, a predição de FA. Estudos recentes mostram que outros parâmetros ecocardiográficos podem se alterar mais precocemente e identificar pacientes com maior risco de FA.^4^ O volume tridimensional do AE e a taxa de *strain* atrial são os dois parâmetros mais estudados neste contexto.^4^

A rigidez arterial já é reconhecida como um preditor independente do desenvolvimento de eventos cardiovasculares maiores, especialmente em pacientes hipertensos.^5^ A velocidade da onda de pulso carotídeo-femoral é considerada o método padrão-ouro para sua avaliação. Grande parte do mecanismo fisiopatológico por trás da rigidez arterial está relacionado à perda de elastina e ao aumento da deposição de colágeno em grandes artérias, um processo que ocorre "naturalmente" com a idade e é acelerado em condições como a HAS. Em uma excelente revisão sistemática sobre o assunto, os mesmos autores do artigo principal fornecem evidências de que esse mesmo mecanismo ocorre no miocárdio e é um determinante tanto para HVE quanto para miocardiopatia atrial.^3^

Dessa forma, é possível estabelecer um continuum entre aumento da pressão arterial, maior rigidez arterial, remodelamento miocárdico, miocardiopatia atrial, aumento da incidência de arritmias atriais e, se o processo não for controlado a tempo, FA.

Nesta edição dos Arquivos Brasileiros de Cardiologia, Lage et al. avaliaram a relação entre alterações funcionais precoces do AE, a presença de rigidez arterial e o aumento da prevalência de arritmias atriais no Holter de 24 horas em um grupo de pacientes hipertensos.^6^ Os autores relatam que o aumento da rigidez arterial está independentemente associado a uma maior densidade de contrações atriais prematuras (CAP), mesmo após ajustes para o índice de massa ventricular esquerda. Esses dados estabelecem uma conexão entre rigidez arterial e comportamento atrial subclínico.

No entanto, alguns desafios e limitações do estudo devem ser destacados. O tamanho da amostra é pequeno, e o estudo é unicêntrico. Além disso, o desenho transversal deste estudo não permite estabelecer uma relação causal entre a rigidez arterial e o aumento de CAPs. Estudos prospectivos maiores são necessários para confirmar esses achados e determinar se há uma causalidade entre a rigidez arterial, alterações atriais e a incidência de FA. Em um estágio posterior, também é necessário avaliar se a população de alto risco se beneficiaria de metas mais rigorosas de controle da pressão arterial ou estratégias farmacológicas específicas.
