# Supplementary material for: Cardiomiopatia Atrial e Hipertensão: Conexões entre Rigidez Arterial e Arritmias Atriais Subclínicas
Source: Arq Bras Cardiol. 2024 Oct 22;121(10):e20240598. [Article in Portuguese] doi: 10.36660/abc.20240598 (PMC11634227; doi:10.36660/abc.20240598)
Supplement: Translation

# Translation: Atrial Cardiomyopathy and Hypertension: Connections between Arterial Stiffness and Subclinical Atrial Arrhythmias

**DOI:** 10.36660/abc.20240598i

**Authors:** Ronaldo Altenburg Gismondi, Mario Fritsch Neves

**Affiliations:** 1 Niterói RJ Brazil Universidade Federal Fluminense - Departamento de Medicina Clínica, Niterói, RJ – Brazil; 2 Rio de Janeiro RJ Brazil Universidade do Estado do Rio de Janeiro - Clinica Medica, Rio de Janeiro, RJ – Brazil

**Keywords:** Hypertension, Vascular Stiffness, Cardiac Arrhythmias

Systemic arterial hypertension (SAH) is the primary risk factor for cardiovascular disease, promoting activation of the renin-angiotensin-aldosterone system (RAAS), endothelial dysfunction, arterial stiffness, fibrosis, and myocardial hypertrophy. Prospective studies show that the consequences include left ventricular hypertrophy (LVH), left atrial (LA) overload, and a higher risk of atrial fibrillation (AF).^1^ In the classic Framingham study, hypertension was an independent predictor of AF and, on its own, accounted for more cases of this arrhythmia than any other risk factor.^1^

The recognition of a "pre-AF" phase, where atrial functional changes have not yet translated into dilation or clinically manifest AF, is of utmost importance in modern cardiology, as early recognition allows for therapeutic interventions before the condition evolves into an established disease. Functional and structural changes in the LA are predictors of atrial arrhythmias during rhythm monitoring, and these, in turn, are predictors of a higher risk of AF.^2,3^

Atrial cardiomyopathy is an increasingly recognized condition identified through various functional tests, and the most used method is echocardiography. In the past, measuring LA volume was the primary measure of atrial dysfunction and, thus, the prediction of AF. Recent studies show that other echocardiographic parameters may alter earlier and identify patients at higher risk for AF.^4^ The LA three-dimensional volume and atrial strain rate are the two most studied parameters in this context.^4^

Arterial stiffness is already recognized as an independent predictor of the development of major cardiovascular events, especially in hypertensive patients.^5^ The carotid-femoral pulse wave velocity is considered the gold standard method for its assessment. Much of the pathophysiological mechanism behind arterial stiffness is related to elastin loss and increased collagen deposition in large arteries, a process that occurs "naturally" with age and is accelerated in conditions like SAH. In an excellent systematic review of the subject, the same authors of the main article provide evidence that this same mechanism occurs in the myocardium and is a determinant both for LVH and atrial cardiomyopathy.^3^

Thus, it is possible to establish a continuum from increased blood pressure, greater arterial stiffness, myocardial remodeling, atrial cardiomyopathy, an increase in the incidence of atrial arrhythmias, and, if the process is not controlled in time, AF.

In this edition of the *Arquivos Brasileiros de Cardiologia*, Lage and colleagues evaluated the relationship between early functional changes in the LA, the presence of arterial stiffness, and the increased prevalence of atrial arrhythmias on 24-hour Holter monitoring in a group of hypertensive patients.^6^ The authors report that increased arterial stiffness is independently associated with a higher density of premature atrial contractions (PAC), even after adjustments for left ventricular mass index. This data establishes a connection between arterial stiffness and subclinical atrial behavior.

However, some challenges and limitations of the study should be highlighted. The sample size is small, and the study is single-center. Additionally, the cross-sectional design of this study does not allow for establishing a causal relationship between arterial stiffness and the increase in PACs. Larger prospective studies are needed to confirm these findings and to determine if there is a causality between arterial stiffness, atrial changes, and the incidence of AF. At a later stage, it is also necessary to evaluate whether the high-risk population would benefit from stricter blood pressure control targets or specific pharmacological strategies.
